# A Review on the Kinetics of Iron Ore Reduction by Hydrogen

**DOI:** 10.3390/ma14247540

**Published:** 2021-12-09

**Authors:** Aidin Heidari, Niusha Niknahad, Mikko Iljana, Timo Fabritius

**Affiliations:** 1 Process Metallurgy Research Unit, University of Oulu, 90570 Oulu, Finland; aidin.heidari@oulu.fi (A.H.); mikko.iljana@oulu.fi (M.I.); 2 Department of Materials Science and Engineering, Sharif University of Technology, Tehran 1458889694, Iran; n.niknahad97@sharif.ir

**Keywords:** hydrogen, green steelmaking, kinetics, ironmaking, iron ore, reduction

## Abstract

A clean energy revolution is occurring across the world. As iron and steelmaking have a tremendous impact on the amount of CO_2_ emissions, there is an increasing attraction towards improving the green footprint of iron and steel production. Among reducing agents, hydrogen has shown a great potential to be replaced with fossil fuels and to decarbonize the steelmaking processes. Although hydrogen is in great supply on earth, extracting pure H_2_ from its compound is costly. Therefore, it is crucial to calculate the partial pressure of H_2_ with the aid of reduction reaction kinetics to limit the costs. This review summarizes the studies of critical parameters to determine the kinetics of reduction. The variables considered were temperature, iron ore type (magnetite, hematite, goethite), H_2_/CO ratio, porosity, flow rate, the concentration of diluent (He, Ar, N_2_), gas utility, annealing before reduction, and pressure. In fact, increasing temperature, H_2_/CO ratio, hydrogen flow rate and hematite percentage in feed leads to a higher reduction rate. In addition, the controlling kinetics models and the impact of the mentioned parameters on them investigated and compared, concluding chemical reaction at the interfaces and diffusion of hydrogen through the iron oxide particle are the most common kinetics controlling models.

## 1. Introduction

Nowadays, carbon dioxide emissions have become one of the most important environmental concerns, the consequences of which include global warming. Approximately 7% of the total carbon dioxide produced in the world belongs to the iron and steel industries [1]. On average, 1.9 tons of carbon dioxide are emitted per ton of crude steel produced. Hence, the iron and steel industries are trying to reduce carbon dioxide emissions and make the iron production process more environmental-friendly by developing new technologies [2]. Among the technologies being developed in this regard, hydrogen as a reducing agent [3,4,5,6], carbon capture and storage (CCS) [7,8], carbon capture and utilization (CCU) [9,10], biomass as a reducing agent [11,12], and electrolysis can be mentioned [13,14,15].

Hydrogen such as carbon monoxide can reduce iron oxides and produces water vapor instead of carbon dioxide. At the moment, a mixture of hydrogen and carbon monoxide is used for iron ore reduction in the direct reduction plants by reforming the natural gas. Hence, it seems by performing some modifications and considering the technical and economical essentials, direct reduction plants can use hydrogen as the only reducing agent in the future. Furthermore, hydrogen can be produced from renewable sources such as biomass that makes the process more environmentally friendly. Therefore, the reduction of iron ore with hydrogen has attracted much attention in recent years [16,17].

In order to minimize the consumption of energy and other elements, it is crucial to study the kinetics of reduction reactions. As there are several reactions that are occurring simultaneously, the kinetics of reduction is very complex [18].

Due to the working temperature, there are different ways to obtain metallic iron from hematite. Reactions can be a chain of single reaction, single and double reaction or a triple reaction as the following [19]: (${\mathrm{Fe}}_{2}O_{3}$, ${\mathrm{Fe}}_{3}O_{4}$ and FeO corresponds to hematite, magnetite and wüstite, respectively).

(1) $${\mathrm{Fe}}_{2}O_{3}\to {\mathrm{Fe}}_{3}O_{4}\to \mathrm{Fe}\;\mathrm{for}\;T<450\;{}^{\circ}C$$

(2) $${\mathrm{Fe}}_{2}O_{3}\to {\mathrm{Fe}}_{3}O_{4}\to {\mathrm{Fe}}_{x}O+\mathrm{Fe}\to \mathrm{Fe}\;\mathrm{for}\;450\;{}^{\circ}C<T<570\;{}^{\circ}C$$

(3) $${\mathrm{Fe}}_{2}O_{3}\to {\mathrm{Fe}}_{3}O_{4}\to \mathrm{FeO}\to \mathrm{Fe}\;\mathrm{for}\;T>570\;{}^{\circ}C$$

**Figure 1:**
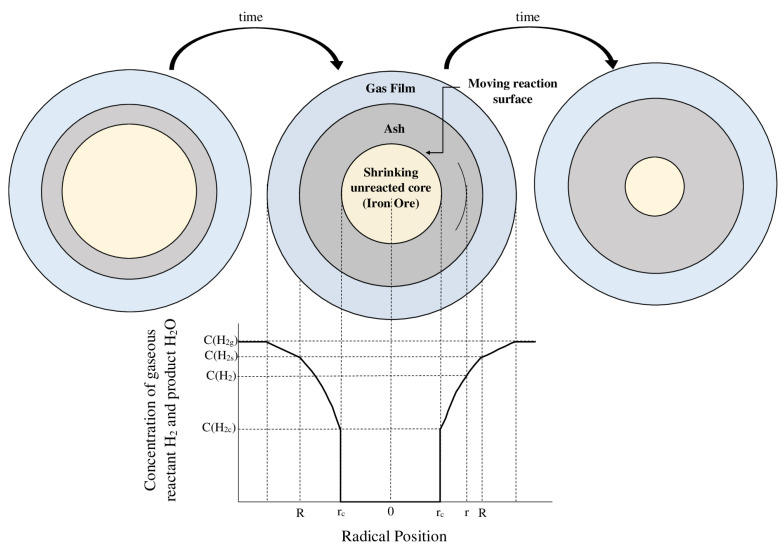
Schematic shrinking core model modified after [21] “modified”.

Due to its nature, the kinetics of iron ore reduction by hydrogen can be classified as gas–solid reactions. There are usually three kinetic-controlling mechanisms of diffusion through the gas film layer, diffusion through the ash layer, and the chemical reaction for these reactions (Figure 1). Hence, five steps can be considered for iron ore reduction by hydrogen [3,20,21]:

1. Diffusion of hydrogen through the film surrounding the iron ore particle.
2. Diffusion of hydrogen through the blanket of ash (consisting of the final product, i.e., iron, and gangue such as silica, alumina, etc.) to the surface of the unreacted iron ore.
3. Chemical reaction of hydrogen with iron ore at this reaction surface.
4. Diffusion of the gaseous product (H_2_O) through the ash back to the exterior surface of the particle.
5. Diffusion of the gaseous product (H_2_O) through the gas film back to the main body of fluid [20,21].

- Control by diffusion through the gas film
  (4) $$\frac{t}{\tau }=X,\;\tau =\frac{\rho R}{3bk_{g}C_{g}}$$
- Control by diffusion through the ash layer
  (5) $$\frac{t}{\tau }=1-3{\left(1-X\right)}^{\frac{2}{3}}+2\left(1-x\right)\;\tau =\frac{\rho R^{2}}{6bD_{e}C_{g}}$$
- Chemical reaction control
  (6) $$\frac{t}{\tau }=1-{\left(1-X\right)}^{\frac{1}{3}},\;\tau =\frac{\rho R}{bk^{\prime \prime }C_{g}}$$

where “*t*” is time, “*X*” is fraction of reacted material (reaction fraction), and “*τ*” is the reaction completion time. “*ρ*” is the density of solid reactant, “*R*” is the radius of the particle, “b” is the stoichiometric coefficient of the solid reactant when the stoichiometric coefficient of the gaseous reactant is equal to 1, “*k*_g_” is the mass transfer coefficient. “*C*_g_” is the concentration of the gaseous reactant in the main body of fluid, “*D*_e_” is the diffusion coefficient, and “*k*″” is the rate constant of the reaction.

For the high gas velocity, it can be assumed that film diffusion does not offer any resistance to transfer and reaction. Thus, film resistance can safely be ignored [20,21].

It can be determined from Equations (5) and (6) that the reduction time is proportional to *R*^2^ and *R* for the diffusion through ash layer and chemical reaction respectively. So, by plotting log (time) vs. log (particle radius), the slope of the line can determine the kinetics controlling mechanism [22].

**Figure 2:**
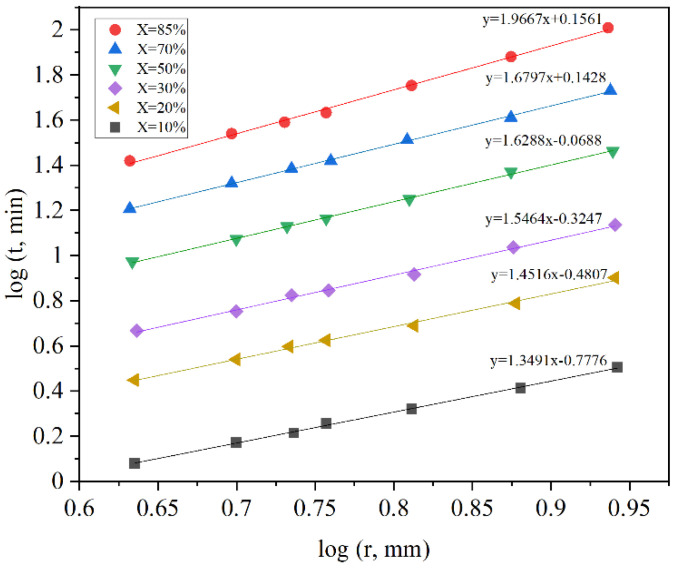
Plot of log (time) vs. log (particle size) for different reaction fractions [22] “modified”.

Figure 2 illustrates that chemical reaction and ash diffusion are both controlling factors, but diffusion limits the rate of reduction mainly near the end. Although several parameters such as temperature, porosity, mineralogy, etc. have an effect on the kinetics of reduction, it seems that the two mentioned mechanisms are the major kinetics controlling mechanisms for the iron reduction by a gaseous reductant [22]. Some researchers also considered the mechanisms based on nucleation and growth.

In this research, the studies that have determined the effect of different parameters on the kinetics of reduction by experiment, modeling and simulation are reviewed.

## 2. Effect of Different Parameters on the Kinetics of Reduction

### 2.1. Effect of Temperature

The effect of temperature on the kinetics of reaction was investigated from two points of view. The dependence of rate constant is explained by the Arrhenius equation (Equation (7)), where “*k*” is the rate constant, “*A*” is the frequency factor, which is related to the frequency of collisions and the orientation of a favorable collision probability, “*E_a_*” is the activation energy, “*R*” is the ideal gas constant, and “*T*” is the temperature in Kelvin.

(7) $$k=A\exp\frac{-E_{a}}{RT}$$

The diffusion coefficient in solids also is a function of temperature and is expressed by the Arrhenius equation (Equation (8)). In this equation, *“D*_0_*”* is the maximal diffusion coefficient, “*E_a_*” is the activation energy of diffusion, “*R*” is the ideal gas constant, and “*T*” is the temperature in Kelvin.

(8) $$D=D_{0}\exp\frac{-E_{a}}{RT}$$

Hence, in both cases, the rate of reduction will increase exponentially by increasing the temperature.

**Figure 3:**
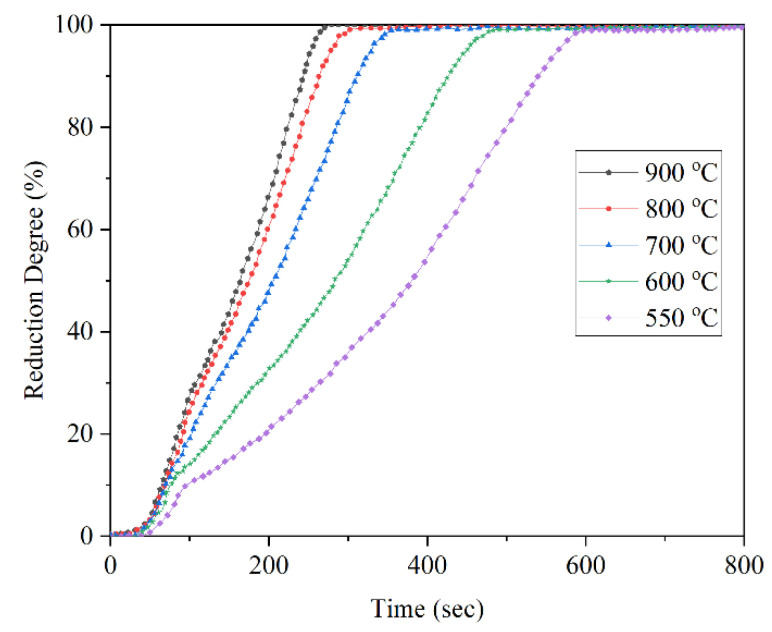
Effect of temperature on the reduction rate (100% H_2_) [26] “modified”.

Barde et al. investigated the reduction rate in the range of 800–1000 °C and found that the increase in reduction rate by increasing the temperature is more effective at the early stages of the reduction [23]. Valipour et al., presented a mathematical model of the reduction of iron ore by a gas mixture of hydrogen, water vapor, carbon monoxide, and carbon dioxide. The results confirmed the positive dependence of temperature on the reduction rate. They found that the increase in diffusivity and reaction rate are the causes of this phenomenon [24]. Tsay et al. also achieved similar results in their experiments and mathematical model. Their results also showed that the higher diffusivity of hydrogen at higher temperatures can overcome the larger gas transport resistance of the larger pellets [25]. Baolin et al. found that for temperatures higher than 600 °C, the reduction of Fe_2_O_3_ to Fe_3_O_4_ is very fast and as a result, the effect of temperature on the reduction rate is not considerable. However, for the reduction of Fe_3_O_4_ to FeO the temperature becomes more effective and increasing the reduction rate can be obviously detected. By the further reduction of FeO to Fe, the effect of temperature becomes negligible again [19]. Wagner et al. reached similar results. Their experiments showed that the effect of temperature on the reduction rate is more intensive for the reduction temperatures lower than 800 °C (Figure 3) [26]. Choi et al., achieved the 80% and 100% reduction at 5 and 10 s, respectively, at 1100 °C [27] while the 80% was obtained in the study by Fruehan et al. in about 500 s at 600 °C, both in the 100% H_2_ flow [28].

### 2.2. Effect of H_2_/CO Ratio

**Figure 4:**
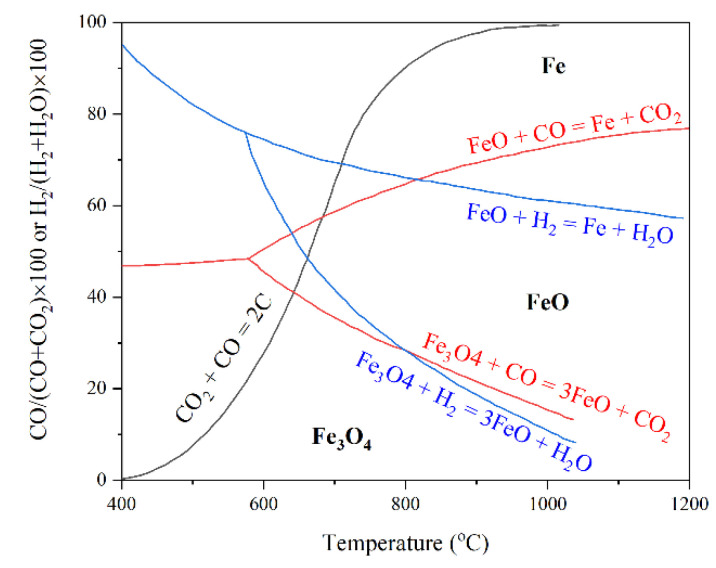
Gas composition in equilibrium with iron and iron oxide phases as a function of temperature [29] “modified”.

The ratio of hydrogen to carbon monoxide in the reducing gas is of great importance to control the reduction rate. The equilibrium diagram of iron oxides, hydrogen and carbon monoxide (Figure 4) shows the equilibrium gas composition to reduce iron ore. Thermodynamic calculations have shown that CO has a higher reducing ability at lower temperatures, whereas reduction by H_2_ is more thermodynamically stable at higher temperatures [29]. From a kinetic point of view, due to the atomic size of hydrogen and its high diffusivity, H_2_ is a faster reductant in comparison with CO at temperatures above 850 °C [30]. Hence, increasing the temperature both thermodynamically and kinetically improves the hydrogen reduction.

**Figure 5:**
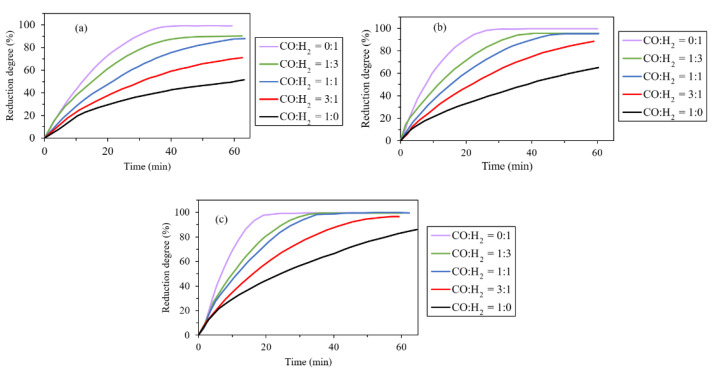
Change of reduction degree with reducing time: (**a**) 800 °C; (**b**) 900 °C; (**c**) 1000 °C [32] “modified”.

Zuo et al. investigated the effect of mixture gas composition on reduction degree and compared the experimental data with a mathematical model. In this study, increasing the reaction rate with the higher hydrogen content in three temperatures (800 °C, 900 °C and 1000 °C) is reported (Figure 5). This occurs due to the higher reducing and diffusing capacities of hydrogen compared with CO at temperatures above 890 °C. Furthermore, as the temperature increases the rise of reduction degree for the higher H_2_ content decreases, which is also approved by Kemppainen et al. [31,32]. In addition, the suggested mathematical model presents an acceptable linear relationship with the reduction degree (over 93%). The only exception is for the cases at 1000 °C, which is justified due to the deformation of pellets at high temperatures [32].

In another study, Yi et al., studied the reduction rate of iron ore pellets at 850, 900, 950, 1000 and 1050 °C with varying H_2_/CO proportions from 0.4 to 2.6. By increasing the ratio range from 0.4 to 1.6, a superior reduction rate was observed. On the other hand, changing the ratio range from 1.6 to 2.6 has little effect on the reduction rate. This indicates the importance of choosing the right ratio of H_2_/CO and not simply raising the amount of hydrogen content [33]. Abdelrahim et al., found that the pellets reduced in CO-CO_2_-H_2_-H_2_O-N_2_ have more porosity and surface area than that reduced in CO-CO_2_-N_2_ [34]. Formation of carbide, slower reduction, and reaching the complete reduction at higher temperatures, for the hematite reduction by CO in comparison with H_2_ was reported by Abu Tahari et al. [35].

**Figure 6:**
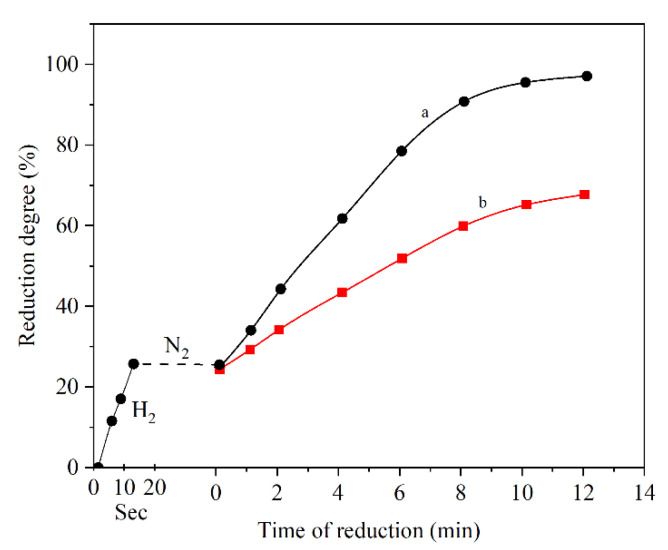
Reduction of wüstite at 1000 °C in (**a**) H_2_ followed by CO; (**b**) pure CO [36] “modified”.

El-Geassy observed the role of hydrogen in the H_2_/CO gas mixture for reducing wüstite. In this experiment, wüstite was reduced up to 25%. The next step was substituting hydrogen with nitrogen for a while and then the reduction continued in pure CO atmosphere. The result of this experiment compared with reducing the wüstite with pure CO from the beginning. Figure 6 designates that using H_2_/CO instead of pure CO accelerates the reduction rate. The main cause of this observation is the nucleating of iron on the wüstite surface in the H_2_ atmosphere. The addition of H_2_ to CO facilitates the nucleation of iron on the surface of the wüstite and also accelerates the growth of iron grains [36].

### 2.3. Effect of Hydrogen Flow Rate

**Figure 7:**
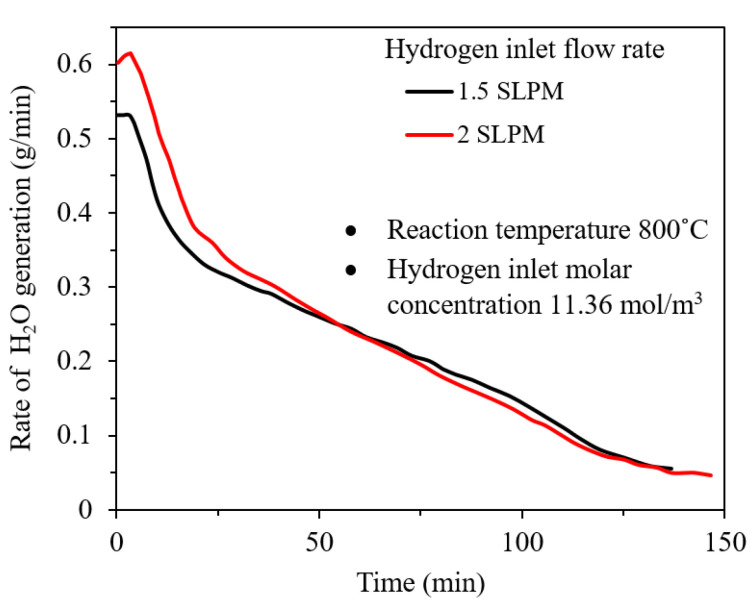
Rate of steam generation during the reduction for a hydrogen inlet flow rate of 1.5 and 2.0 SLPM at 800 °C [23] “modified”.

The hydrogen inlet flow rate can specify the overall concentration of hydrogen in the reactive structure. Barde et al. studied the reduction of iron–silica Magnetically Stabilized Porous Structure (MSPS) by hydrogen. Figure 7 shows the steam generation for 1.5 and 2 standard liters per minute hydrogen inlet flow rate at 800 °C. It was observed that a higher inlet flow rate results in higher steam generation at the early stages of the reaction. However, the two graphs are not notably different at the later stages [23]. Kawasaki et al. realized that there is a critical gas velocity, which is specified experimentally at different temperatures. The superficial gas velocity considered 0.01 to 0.03 mol/(min.cm^3^). Below critical gas velocity, the gas flow rate controls the rate of the reaction [37].

**Figure 8:**
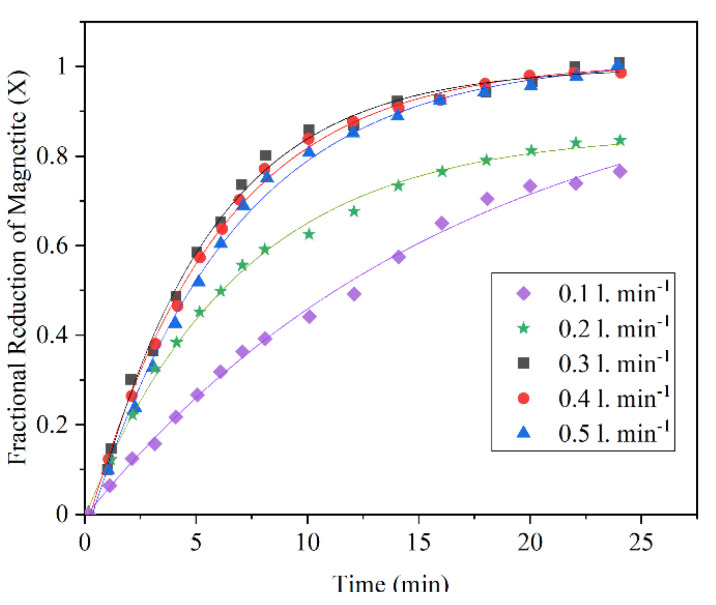
Fractional reduction of magnetite vs. time plot for the reduction of magnetite ore fines at different flow rates at 900 °C [38] “modified”.

Kulia et al. investigated the effect of various hydrogen flow rates (0.1, 0.2, 0.3, 0.4, and 0.5 L/min) on fractional reduction of magnetite ore at 900 °C and 1 atm pressure. The result of the experiments shows an increase in fractional reduction as the flow rate raises from 0.1 to 0.4 L/min (Figure 8). In addition, the flow rates above 0.4 L/min are not noticeably different from the others. Therefore, 0.4 L/min was considered as the optimum hydrogen flow rate [38].

Ohmi et al., evaluate the effect of hydrogen flow rate, which was diluted by N_2_/H_2_O on the mixed control plots. The conclusion is similar to [32]. Furthermore, resistance for gaseous diffusion around a pellet increases with the decrease in gas flow rate due to experiments [39,40].

### 2.4. Effect of Mineralogy

**Figure 9:**
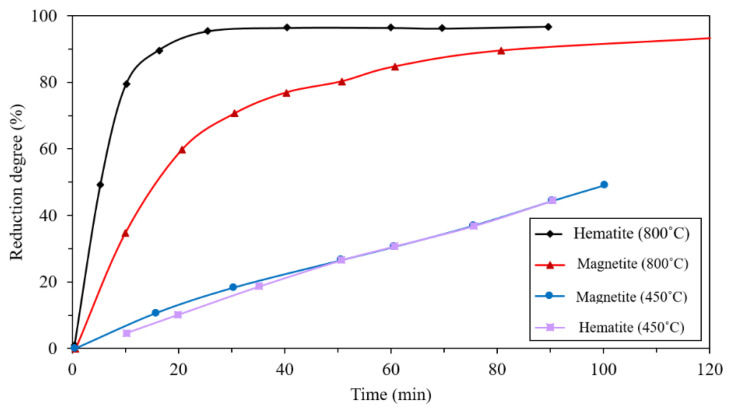
Comparison of hematite and magnetite reduction at the two temperatures 450 °C and 800 °C [41] “modified”.

**Figure 10:**
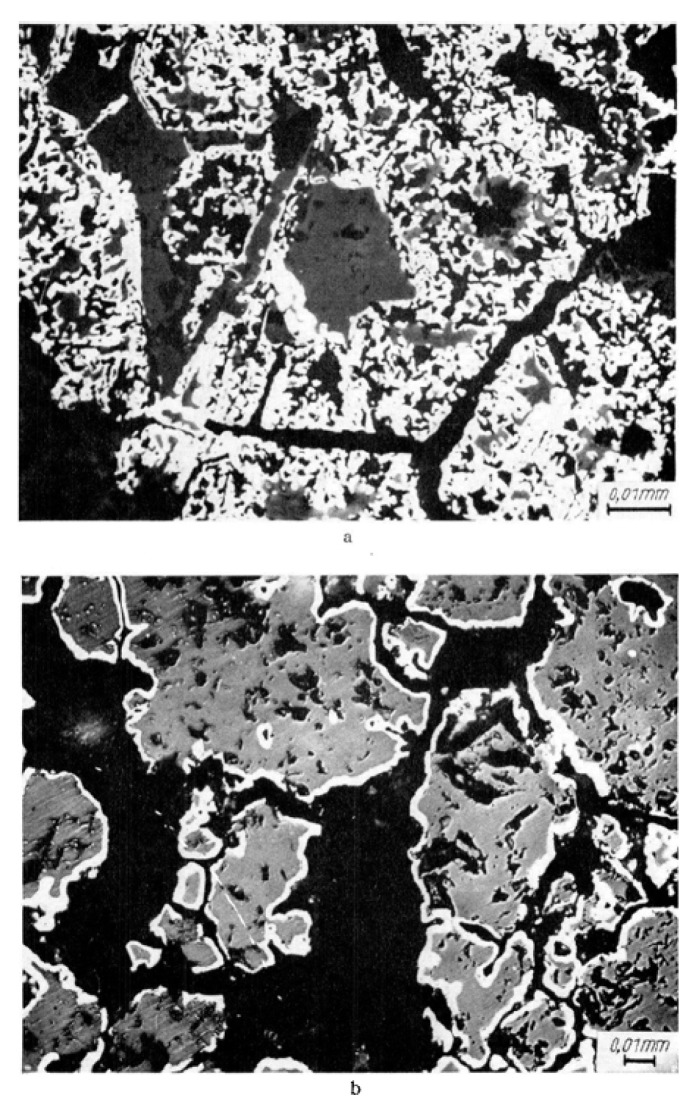
The SEM images of (**a**) reduced hematite; (**b**) reduced magnetite [43].

**Figure 11:**
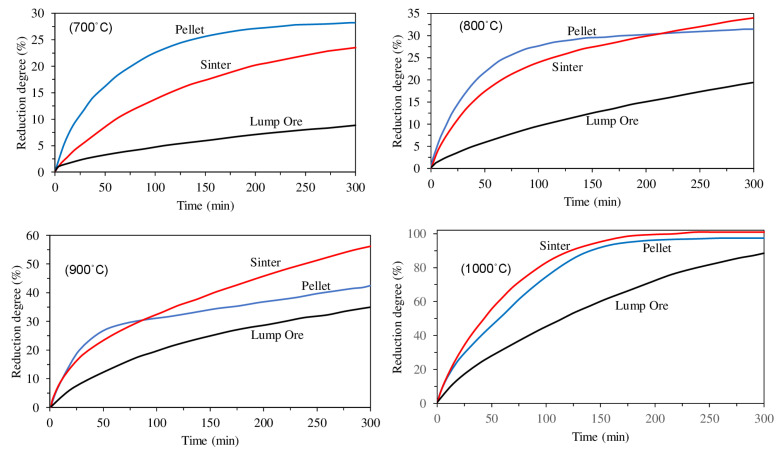
Reduction degree as a function of time for iron ore pellet, sinter, and lump ore at 700, 800, 900, and 1000 °C [44] “modified”.

Edstrom et al., compared hydrogen reduction of hematite with magnetite. As illustrated in Figure 9, the reduction of hematite by hydrogen is faster than the reduction of magnetite, especially at higher temperatures. This is because of the hard and dense shell of magnetite, which causes lower diffusivity [41]. Furthermore, due to the higher density of hematite (5.260 g/cm^3^) in comparison with magnetite (5.175 g/cm^3^), during the reduction of hematite to magnetite, some microcracks form because of the volume change. The formed cracks work as porosities and make the diffusion of gas easier (Figure 10) [42,43]. Heikkilä et al. compared the reduction behavior of iron ore pellets, sinter, and lump ore at different temperatures. The lump ore showed the lowest reduction rate at all temperatures due to its low porosity and surface area. At the lower temperatures, (lower than 800 °C) the pellet reacted faster, but at the higher temperatures, the reduction rate of the sinter was higher (Figure 11). It was found that it is because of the higher initial magnetite content in the composition of the sintered sample, which reduces slowly at low temperatures [44].

Oxy-hydroxides such as goethite have shown high reducibility due to the high surface area caused by water loss [45].

**Figure 12:**
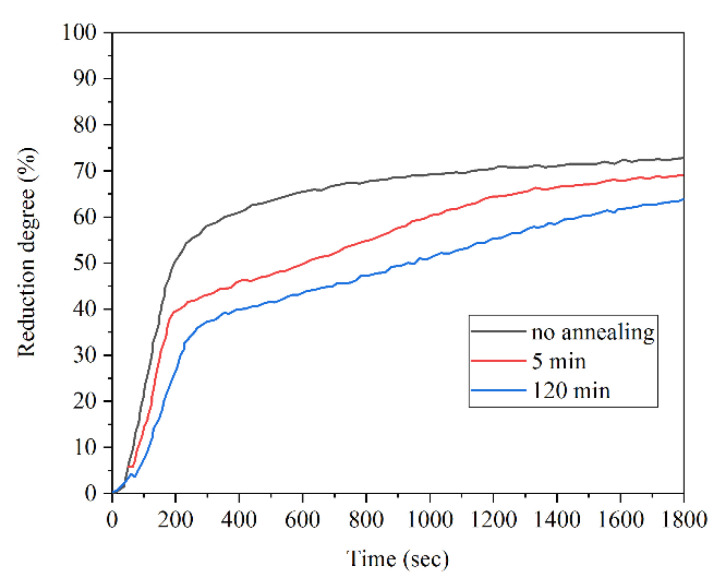
Effect of annealing on the reduction of Wabush ore at 800 °C in 60% H_2_ [28] “modified”.

Fruehan et al. found that if iron oxides are converted to magnetite and kept in the same form as magnetite for a few minutes before reduction to iron, which can be called “annealing”, it causes a decrease in the degree of reduction especially at higher temperatures and lower hydrogen pressures (Figure 12) [28].

### 2.5. Effect of Particle Size

**Figure 13:**
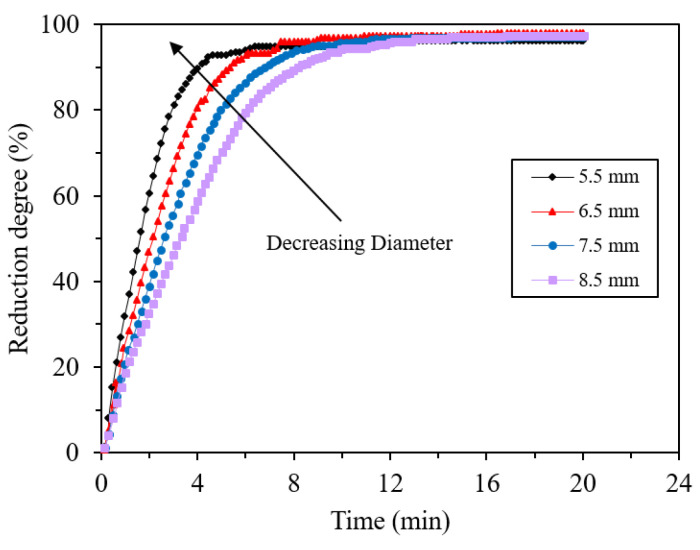
Dependence of reduction degree on reduction time of oxidized spherical pellets with different diameters. Data obtained at 1070 °C in flowing H_2_ gas [46] “modified”.

Zhang et al. found that by increasing the pellet size from 5.5 to 8.5 mm, the reduction rate decreases steadily due to the shorter diffusion distance (Figure 13) [46]. Similar results were obtained by researchers [47,48,49], which were approved by mathematical modeling [24]. At low reduction temperatures, particle size is not an effective parameter and other rate-limiting parameters become more important [50]. Hou et al. studied the reduction of particle sizes from 0.025 to 0.2 mm. The results showed that for the particle sizes smaller than 0.045 mm the dependence of reduction rate on particle size is not considerable, because the internal diffusion resistance is neglectable for particle size below 0.045 mm [19].

**Figure 14:**
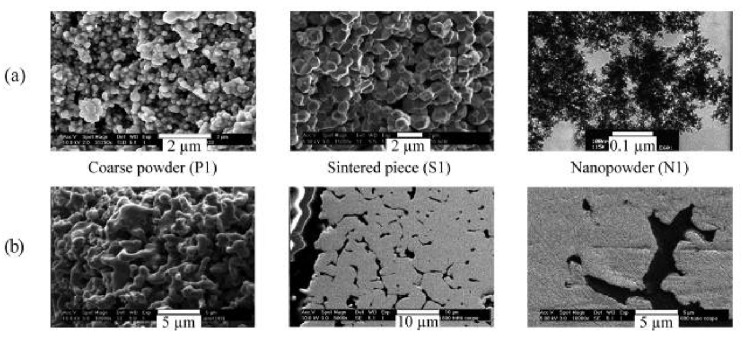
Cross-sections of the different samples (**a**) before and (**b**) after reduction [26].

Wagner et al. investigated the hydrogen reduction of three samples of coarse powder (P1), sintered piece (S1), and nanopowder (N1). Although the specific area for N1 and S1 is higher than P1 due to their smaller particle size, the reduction of the P1 sample was faster than the two other samples. By investigation of the reduced samples’ morphology, it became clear that S1 and N1 samples became compact and lost most of their porosities after reduction while the P1 sample retains its porosity after the reduction (Figure 14) [26].

### 2.6. Effect of Impurities

**Figure 15:**
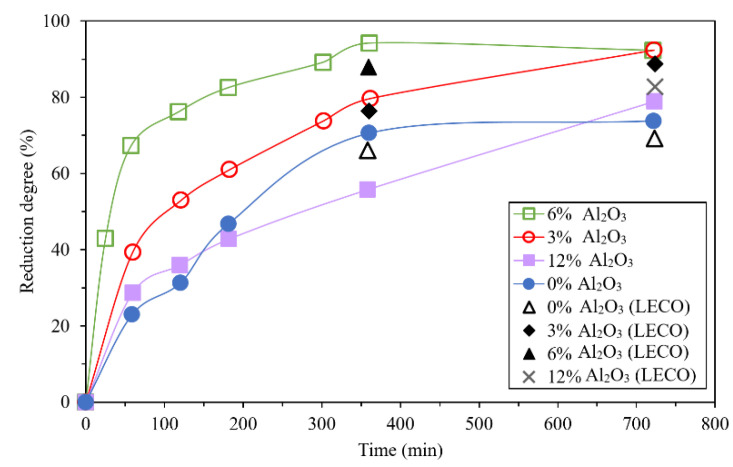
Effect of alumina content on reduction of Fe_3_O_4_–Al_2_O_3_ system at 850 °C by CO–CO_2_ gas mixture (80% CO) [52] “modified”.

**Figure 16:**
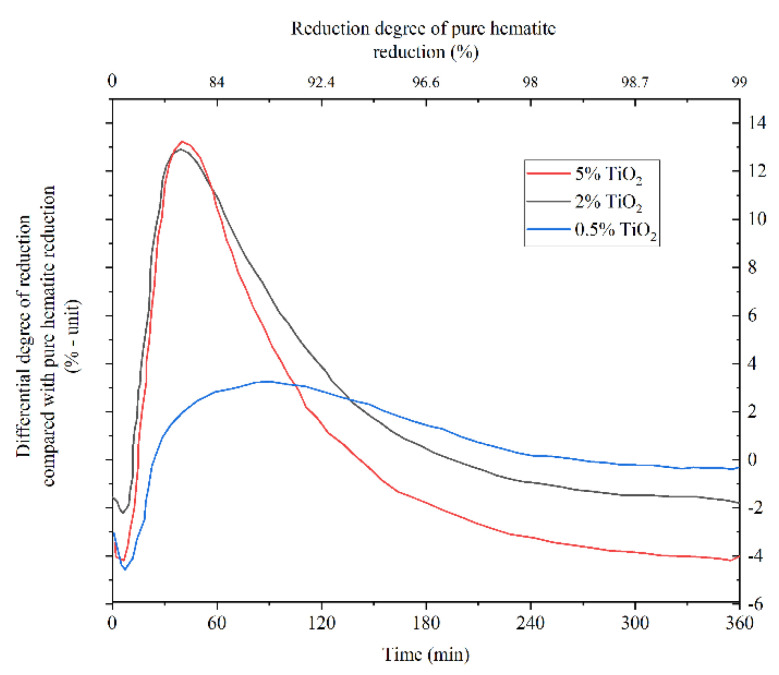
Effect of titanium dioxide on reduction of hematite. The curves show the difference from the reduction curve of pure hematite. The grade of reduction for pure hematite can be read from the secondary x-axis at the top of the figure [53] “modified”.

The effect of impurities on the reduction of iron ore by hydrogen is mostly similar to the reduction of carbon monoxide. Qie et al. found that by increasing the temperature and hydrogen concentration, the formation of the phases such as MgFe_2_O_4_ and Fe_x_Si_y_O_4_ become faster, which increases the resistance of interfacial chemical reaction during the reduction. Higher contents of CaO, SiO_2_, and MgO can be found in larger particles which leads to the formation of cracks and accelerate the reduction of wüstite [51]. Alumina forms Fe_3_O_4_–FeAl_2_O_4_ solution by diffusion of Al^3+^ from wüstite, which enriches the hercynite content in the solution at the reaction interface. Further reduction of Fe_3_O_4_–FeAl_2_O_4_ solution leads to the formation of micro-cracks, which increases the rate of reduction (Figure 15) [52]. However, in another research, Teplov found that the presence of Al_2_O_3_ and MgO decreases the rate of magnetite reduction by hydrogen and the effect of Al_2_O_3_ is higher than MgO [47]. The presence of TiO_2_ of more than 0.5 wt.% increases the rate of reduction at the beginning of the reduction process due to the formation of cracks in pellets (Figure 16) [53].

### 2.7. Apparent Activation Energy

Activation energy is the minimum energy required to perform a chemical reaction, or in other words, the energy required to overcome a potential barrier. As mentioned earlier, the dependence of constant rate on the activation energy is expressed by the Arrhenius equation. In the case of complex reactions, the calculated activation energy is actually the average of all elementary steps. However, for an elementary reaction, a spectrum of individual collisions is related to billions of molecules with different geometries, angels, and frequencies of vibration [54].

Researchers have used several methods based on the Arrhenius equation for determining the activation energy. The fraction of reaction is defined as:

(9) $$\alpha =\frac{m_{i}-m}{m_{i}-m_{f}}$$

where *m_i_* is the initial mass of the solid reactant (iron oxide), *m* is the actual mass of solid reactant at the time of *t*, and *m_f_* is the final mass of the solid reactant at the end of reaction or in other words, all of the solid mass that can react in the reaction.

The rate equation for a gas–solid reaction is defined generally as:

(10) $$\frac{d\alpha }{dt}=k\left(T\right)\times f\left(\alpha \right)$$

where *k(T)* is the rate constant as a function of temperature, and *f(α)* is a function of the fraction of reaction that depends on the kinetics model. Using the Arrhenius equation:

(11) $$\frac{d\alpha }{dt}=A\times \exp\left(-\frac{E_{a}}{RT}\right)\times f\left(\alpha \right)$$

(12) $$\ln\left(\frac{\frac{d\alpha }{dt}}{f\left(\alpha \right)}\right)=-\frac{E_{a}}{RT}+\ln\left(A\right)$$

**Figure 17:**
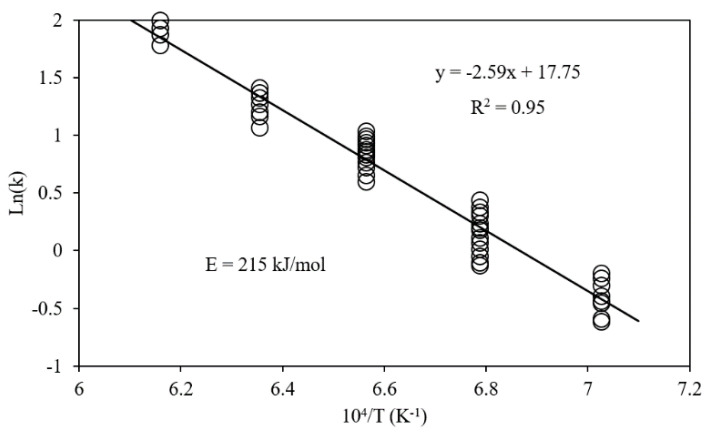
Arrhenius plot between Ln *k* and 10^4^/*T*. (k in atm^−1^/s). Points at each temperature represent data under different H_2_ partial pressures and reaction times [48] “modified”.

By plotting the $\ln\left(\frac{\frac{d\alpha }{dt}}{f\left(\alpha \right)}\right)$ versus $\frac{1}{T}$ the activation energy can be determined from the slope of the line [10,19,23,41,49,51,55,56,57,58] (Figure 17).

Some researchers used the Kissinger method for calculating the activation energy [59,60]. In this method, the maximum rate of reaction is achieved when the derivation of the equation with respect to time is zero:

(13) $$\frac{d}{dt}\left(\frac{d\alpha }{dt}\right)=\frac{d}{dt}\left(A\times \exp\left(-\frac{E_{a}}{RT}\right)\times f\left(\alpha \right)\right)=0$$

After derivation, Equation (14) is obtained:

(14) $$A\times \frac{E_{a}}{RT^{2}}\times \exp\left(-\frac{E_{a}}{RT}\right)\frac{dT}{dt}\times f\left(\alpha \right)+A\times \exp\left(-\frac{E_{a}}{RT}\right)\times \frac{df\left(\alpha \right)}{dt}=0$$

Using “Chain rule”, Equation (15) can be expressed as:

(15) $$\frac{df\left(\alpha \right)}{dt}=\frac{df\left(\alpha \right)}{d\alpha }\times \frac{d\alpha }{dt}$$

The *g*(*α*) and *Φ* can be defined as:

(16) $$\frac{d\alpha }{df\left(\alpha \right)}=g\left(\alpha \right)$$

(17) $$\frac{dT}{dt}=\phi$$

**Figure 18:**
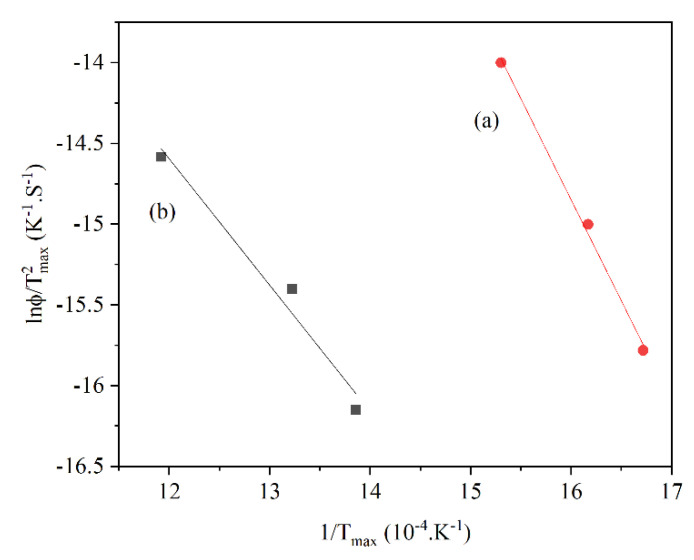
Temperature-programmed Arrhenius plots for the two-step reduction. (**a**) Fe_2_O_3_ → Fe_3_O_4_, (**b**) Fe_3_O_4_ → Fe [60] “modified”.

Using Equations (11) and (14)–(17), Equation (13) can be further rewritten as:

(18) $$\ln\left(\frac{\phi }{T_{m}{}^{2}}\right)=\ln\left(\frac{AR}{E_{a}g\left(\alpha \right)}\right)-\frac{E_{a}}{RT_{m}}$$

where *Φ* is the heating rate and *T_m_* is the temperature when the reaction rate is maximum. By plotting $\ln\left(\frac{\phi }{T_{m}{}^{2}}\right)$ versus $\frac{1}{T_{m}}$ the activation energy can be determined from the slope of the line (Figure 18) [61].

The amounts of apparent activation energy determined in various studies related to the reduction of iron oxides to hydrogen are listed in Table 1. As it turns out, the activation energies have a wide range of values. This is because the value of activation energy depends on the chemical composition, physical properties of materials, temperature range, and process conditions. It is inferred from Table 1 that, the activation energy decreases by increasing the percentage of hydrogen in the reducing gas, which indicates the easier reduction of iron oxides by hydrogen than carbon monoxide. In addition, the activation energy for the reduction of natural magnetite to iron is higher than the reduction of magnetite, which has produced by the reduction of hematite. Furthermore, the activation energy is lower for the higher temperature ranges and some researchers reported a decrease in activation energy around a transition temperature that may be related to the changes in the kinetics controlling mechanism.

### 2.8. Kinetics Controlling Models

The summary of some research on the iron oxides reduction by hydrogen and the used kinetics models are provided in Table 2. As was mentioned earlier, the kinetics model depends on the reduction condition such as temperature, iron oxide type, particle size, etc. The table shows that although some researchers have chosen the nucleation models as the controller, chemical reaction at the interfaces and diffusion of hydrogen through the iron oxide particle are two common models among the related studies and it seems that the kinetics of iron ore reduction by hydrogen is mixed-control. Furthermore, it was asserted by most of the researchers that the rate-controlling step is the reduction of wüstite to iron when a dense shell of iron forms on the wüstite layer and the diffusion of hydrogen through this shell becomes difficult [17]. In addition, it should be observed that increasing the temperature improves the kinetics of reduction in both models, but its effect is more significant for diffusion. Hence, at high temperatures and especially at the early stages of the reduction, diffusion is not the rate controller [23,26].

## 3. Conclusions

In order to reduce the amount of CO_2_ pollution in the iron and steel industry, it is crucial to investigate new methods of iron oxide reduction. Among all these techniques, using hydrogen as a reducing agent is receiving much attention. In addition, it is necessary to study the exact material and energy consumption by investigating the kinetics of the reduction reactions. These solid–gas reactions are complex and can vary due to the working temperature, gas atmosphere, chemical composition, etc. The mechanisms that control the kinetics of reduction are the diffusion of H_2_/H_2_O through the gas film layer, diffusion of H_2_/H_2_O through the ash layer, and chemical reaction. For the high gas velocity, film resistance can be ignored. It was approved by some researchers that nucleation also can be considered as a controlling mechanism at the early stages of the reduction and at low temperatures.

Moreover, the parameters such as temperature, porosity, mineralogy, etc. can play a vital role in order to determine the kinetics of hydrogen reduction precisely. The following conclusions can be made for each parameter according to this literature review:

- Effect of Temperature: Due to the Arrhenius equation, by increasing the temperature, the rate of reduction will increase exponentially. At temperatures above 590 °C, the effect of temperature on the reduction of Fe_2_O_3_ to Fe_3_O_4_ and reduction of FeO to Fe is negligible, but for the reduction of Fe_3_O_4_ to FeO it is considerable.
- Effect of H_2_/CO ratio: The reaction rate would increase with the higher hydrogen content at temperatures above 1000 °C. Additionally, H_2_/CO proportion has the most beneficial effect on the reduction rate when being 1.6, and the higher ratios effect is negligible.
- Effect of hydrogen flow rate: Higher inlet flow rate causes higher steam generation at the early stages of the reaction and at the later stages, the effect is minor. Additionally, there is a critical gas velocity below which gas flow rate controls the rate of the reaction.
- Effect of iron ore mineralogy: Because of the hard and dense shell of magnetite in comparison with hematite, magnetite has lower diffusion. Thus, the reduction of hematite by hydrogen is faster than the reduction of magnetite, especially at higher temperatures.
- Effect of particle size: As the size of the particle decreases the specific area increases, therefore the reduction rate enlarges because the reaction starts from the surface. Furthermore, the smaller particle size leads to a shorter distance that gas has to pass to reach inner layers. However, as the particle size shrinks, the chance of agglomeration will increase and as a result, the specific area decreases.
- Effect of impurities: The effect of impurities can be assumed as reduction by H_2_ and CO. Impurities such as CaO, SiO_2_, and MgO and alumina forms can lead to the formation of micro-cracks that promote the reduction of wüstite. Contrarily, some impurities such as Al_2_O_3_ and MgO decrease the rate of magnetite reduction.

Activation energy definition is the minimum energy that is required to perform a chemical reaction. Lower activation energy shows easier reduction of iron oxides. For instance, the activation energy decreases by increasing the percentage of hydrogen in the reducing gas. In fact, activation energy is dependent on chemical composition, physical properties of materials, temperature range, and process conditions. As a result, there is a wide range of values for the activation energy of reactions. The activation energy of natural magnetite to iron is higher than the reduction of the other forms of iron oxide.

Kinetics models depend on different parameters of reduction such as temperature, iron oxide type, particle size, etc. commonly, chemical reactions at the interfaces and the diffusion of hydrogen through the iron oxide particle are used as kinetics models. Through the reduction of wüstite to iron an iron shell covers the surface of the sample and makes the diffusion hard. Thus, wüstite reduction is the rate-controlling and slowest step.

## Figures and Tables

**Table 1:** The amounts of apparent activation energy related to the reduction of iron oxides by hydrogen.

Reference	Reduction Reaction/Step	E_a_ (kJ/mol)	Relevant Operating Conditions
[62]	Fe_2_O_3_ → Fe	57.1	Pure Fe_2_O_3_
	Fe_2_O_3_ → Fe	110.5	Fe_2_O_3_ mixed with MgO
	Fe_2_O_3_ → Fe	108.4	Fe_2_O_3_ mixed with Al_2_O_3_
	Fe_2_O_3_ → Fe	108.4	Fe_2_O_3_ mixed with In_2_O_3_
	Fe_2_O_3_ → Fe	108.4	Fe_2_O_3_ mixed with Li_2_O
	Fe_2_O_3_ → Fe	130.0	Fe_2_O_3_ mixed with TiO_2_
	Fe_2_O_3_ → Fe	89.9	Hematite ore
[60]	Fe_2_O_3_ → Fe_3_O_4_	89.1	5% H_2_ + 95% N_2_
	Fe_3_O_4_ → Fe	70.4	5% H_2_ + 95% N_2_
[63]	Fe_2_O_3_ → Fe	51.0	Hematite ore
	Fe_2_O_3_ → Fe	96.1	Natural single crystals
[64]	Fe_2_O_3_ → Fe	20–46	Fe_2_O_3_ nanopowder
[65]	Fe_2_O_3_ → Fe	15–20	Fe_2_O_3_/metal Pellets
[58]	Fe_2_O_3_ → Fe_3_O_4_	75.9	
	Fe_2_O_3_ → Fe_3_O_4_	94.8	10% H_2_ + 90% N_2_
	Fe_3_O_4_ → Fe	88.0	
	Fe_3_O_4_ → Fe	103.0	10% H_2_ + 90% N_2_
[55]	Fe_2_O_3_ → Fe	28.1	10% H_2_ + 90% N_2_
	Fe_2_O_3_ → Fe	93.7	5.7% CO + 4.3% H_2_ + 90% N_2_
[56]	Fe_2_O_3_ → Fe	111	Hematite pellet with biomass
	Fe_2_O_3_ → Fe	122	Hematite pellet without biomass
[23]	Fe_3_O_4_ → FeO	47	
	FeO → Fe	30	
[66]	Fe_3_O_4_ → Fe	200	227 °C < T < 250 °C
	Fe_3_O_4_ → Fe	71	250 °C < T < 390 °C
	Fe_3_O_4_ → Fe	44	T > 390 °C
[67]	Fe_3_O_4_ → Fe (step)	59–69	5% H_2_ + 95% He
	Fe_3_O_4_ → Fe	61–75	5% H_2_ + 95% He
[19]	Fe_3_O_4_ → FeO	13.5	5% H_2_ + 95% Ar
[57]	Fe_2_O_3_ → Fe	37.4	25% H_2_ + 75% CO
	Fe_2_O_3_ → Fe	40.1	50% H_2_ + 50% CO
	Fe_2_O_3_ → Fe	54.3	75% H_2_ + 25% CO
	Fe_2_O_3_ → Fe	53.5	100% H_2_
[51]	Fe_2_O_3_ → Fe	50.9	5% H_2_ + 30% CO + 65% N_2_
	Fe_2_O_3_ → Fe	36.3	10% H_2_ + 30% CO + 60% N_2_
	Fe_2_O_3_ → Fe	35.8	15% H_2_ + 30% CO + 55% N_2_
	Fe_2_O_3_ → Fe	30.4	20% H_2_ + 30% CO + 50% N_2_
[25]	Fe_2_O_3_ → Fe_3_O_4_	92.0	
	Fe_3_O_4_ → FeO	71.1	
	FeO → Fe	63.6	
[48]	Fe_2_O_3_ → Fe	215	
[36]	FeO → Fe	53.7	100% H_2_
	FeO → Fe	60.6	75% H_2_ + 25% CO
	FeO → Fe	64.8	50% H_2_ + 50% CO
[59]	Fe_2_O_3_ → Fe_3_O_4_	105–120	Fe_2_O_3_ nanopowder
	Fe_3_O_4_ → Fe	55–45	Fe_2_O_3_ nanopowder

**Table 2:** Kinetics models of the iron oxides reduction by hydrogen due to the condition.

Reference	Kinetics Controller	Condition/Description
[62]	topo chemical reaction	Pure Fe_2_O_3_
[60]	two-dimensional nucleation	reduction of hematite to magnetite
[49]	diffusion through ash	
	chemical reaction	
[46]	chemical reaction	reduction of magnetite
[58]	Two- and three-dimensional nucleation	T < 420 °C
	chemical reaction	T > 420 °C
[55]	Two-dimensional nucleation and chemical reaction	initial stage
	diffusion through ash	end of reaction
[56]	chemical reaction	reduction of wüstite
[23]	chemical reaction	
[66]	diffusion	reduction of magnetite at low temperature
[57]	chemical reaction	
	diffusion through ash	
[51]	chemical reaction	
	diffusion through ash	
[65]	chemical reaction	reduction of hematite to magnetite
	diffusion through ash	reduction of magnetite to wüstite
[36]	chemical reaction	reduction of wüstite to iron
[59]	nucleation	reduction of hematite to magnetite
[68]	nucleation	reduction of wüstite to iron
[33]	chemical reaction	reduction of hematite to magnetite
	chemical reaction	reduction of magnetite to wüstite
	diffusion through ash	reduction of wüstite to iron
[69]	chemical reaction	
	diffusion through ash	
[37]	diffusion through film	
	diffusion through ash	
[25]	diffusion through ash	
[70]	nucleation	initial stage
	chemical reaction and diffusion through ash	end of reaction
[48]	nucleation	initial stage
[50]	diffusion through film	reduction of hematite to magnetite
	chemical reaction	reduction of magnetite to wüstite
[28]	diffusion through ash	reduction of wüstite to iron
[71]	diffusion through ash	
[72]	chemical reaction	
	diffusion through ash	

## Data Availability

No new data were created or analyzed in this study. Data sharing is not applicable to this article.
